# Improving uptake and completion of pulmonary rehabilitation in COPD with lay health workers: feasibility of a clinical trial

**DOI:** 10.2147/COPD.S188731

**Published:** 2019-03-12

**Authors:** Patrick White, Gill Gilworth, Simon Lewin, Lauren Hogg, Rachel Tuffnell, Stephanie J C Taylor, Nicholas S Hopkinson, Nicholas Hart, Sally J Singh, Alison J Wright

**Affiliations:** 1 Department of Public Health and Primary Care, School of Population Health and Environmental Sciences, King’s College London, London, UK, patrick.white@kcl.ac.uk; 2 Norwegian Institute of Public Health, Oslo, Norway; 3 Health Systems Research Unit, South African Medical Research Council, Cape Town, South Africa; 4 Physiotherapy Department, Guy’s and St Thomas’ NHS Foundation Trust, London, UK; 5 The Pulmonary Rehabilitation and Integrated Respiratory Team, King’s College Hospital NHS Foundation Trust, London, UK; 6 Centre for Primary Care and Public Health, Blizard Institute, Barts and The London School of Medicine and Dentistry, Queen Mary University of London, London, UK; 7 National Heart and Lung Institute, Imperial College London, London, UK; 8 Lane Fox Respiratory Service, Guy’s and St Thomas’ NHS Foundation Trust, London, UK; 9 Centre for Exercise and Rehabilitation Science, University Hospitals of Leicester NHS Trust, Leicester, UK; 10 Department of Clinical, Educational and Health Psychology, University College London, London, UK

**Keywords:** uptake, completion, recruitment, retention, intervention fidelity

## Abstract

**Purpose:**

This study was designed to evaluate the feasibility of a cluster randomized controlled trial to test the efficacy of lay health workers (LHWs) in improving the uptake and completion of pulmonary rehabilitation (PR) in the treatment of COPD.

**Materials and methods:**

LHWs, trained in confidentiality, role boundaries, and behavior change techniques, supported patients newly referred for PR. Interactions between LHWs and participants were recorded with smartphones. Outcomes were recruitment and retention rates of LHWs, questionnaire and interview-evaluated acceptability and analysis of intervention fidelity.

**Results:**

Forty (36%) of 110 PR-experienced COPD patients applied to become LHWs. Twenty (18%) were selected for training. Twelve (11%) supported patients. Sixty-six COPD patients referred for PR received the intervention (5.5 participants per LHW). Ten LHWs were retained to the end of the study. Seventy-three percent of supported patients were satisfied or very satisfied with the intervention. LHWs delivered the intervention with appropriate style and variable fidelity. LHWs would welcome more intensive training. Based on this proof of concept, a cluster randomized controlled trial of an LHW intervention to improve uptake and completion of PR is feasible.

**Conclusion:**

PR-experienced COPD patients can be recruited, trained, and retained as LHWs to support participation in PR, and can deliver the intervention. Participant COPD patients found the intervention acceptable. A cluster randomized controlled clinical trial is feasible.

## Introduction

There is strong evidence that pulmonary rehabilitation (PR) is effective across the symptoms and disability of COPD and improves health status and quality of life.^1^ PR is “a comprehensive intervention based on … exercise training, education, and behavior change, designed to improve the physical and psychological condition of people with chronic respiratory disease”.^2^ It is recommended in national and international guidelines for people who are functionally impaired by COPD.^2^ In many settings, access to PR is inadequate.^3^ In a UK national COPD audit, only 15% of COPD patients eligible for PR were actually referred.^4^ Where PR is available, its effectiveness is limited by poor uptake and completion. In London, UK, only 40% of 1,111 COPD patients referred to PR completed a PR course.^5^ This completion rate is consistent with that in similar services elsewhere in the UK and in Europe.^3^ As a group, COPD patients are hard to convince that exercise with their peers is an effective treatment.^6^,^7^ In a recent joint statement, the American Thoracic Society and the European Respiratory Society have called for more funding and improved collaboration between clinicians, patients, and funders to achieve better access to PR.^3^ Holland and Cox recently commented about PR that “…until access and uptake are improved, it cannot be considered a successful treatment”.^8^

A systematic review of the obstacles to PR uptake and completion identified disruption to valued routines, uncertainty among referrers regarding the effectiveness of PR, inconvenient timing, travel issues, and lack of perceived benefit as key issues.^9^ The features found to predict consistently noncompletion were being a current smoker and comorbidities, particularly depression. In a qualitative study of participants who had recently completed PR, smoking was associated with feelings of unworthiness to participate.^7^ The suitability of group activity and health professionals’ views of PR were also influential. The study participants would have welcomed the help of patients experienced in PR to introduce them to the treatment and to support them through it. In a recent systematic review, interventions to improve the uptake and completion of PR did not influence the outcome.^10^

The focus of this study, lay health workers (LHWs; sometimes called Community Health Workers or Community Health Volunteers), have been defined as “any health worker carrying out functions related to healthcare delivery, trained in some way in the context of the intervention, and having no formal professional or para-professional certificate or tertiary education degree”.^11^ LHWs are effective in the support of therapeutic interventions in many health settings including depression, diabetes, immunization, hypertension, and maternal and child health.^11^–^14^ We are not aware of studies of the use of LHWs to support the uptake of PR. The advantages of using LHWs to assist in the implementation of evidence-based interventions include shared social backgrounds with patients and shared personal experience of the health issue being targeted.^15^ The high regard that many successful completers of the treatment have for PR suggested that they may be good candidates for the LHW role. The intervention proposed aims to address factors leading to poor uptake and completion after patient referral.

This study was designed to test the feasibility of conducting a cluster randomized controlled trial of the efficacy of LHWs in improving uptake and completion of PR in COPD. Three elements of feasibility were tested: recruitment, training, and retention of PR-experienced COPD patients as LHW volunteers; recruitment of COPD patients, newly referred to PR, to receive LHW support and to report on its acceptability; delivery of the LHW intervention and its fidelity.

## Materials and methods

In this feasibility study completed in south London, UK, we recruited and trained COPD patients experienced in PR to carry out a new LHW role. The role was devised to promote uptake and completion of PR in newly referred COPD patients. We also recruited newly referred COPD patient-participants to receive the intervention and to report on its acceptability.

### Study participants

#### Lay health workers

Inclusion criteria: diagnosis of COPD; completion of PR in the previous 18 months; >40 years; competent in English; independently mobile; able to use a smartphone; willing to undertake LHW training; willing to support up to eight newly referred COPD patients over 6 months; willing to make digital recordings of all patient contacts. Exclusion criteria: current life-threatening illness; serious mental illness.

#### Patient-participants

Inclusion criteria: a diagnosis of COPD; eligibility for PR treatment; and fluency in English. Exclusion criterion: significant other physical or mental health problems that would interfere with participation.

Written informed consent was obtained from all participants. Ethical approval was provided by NRES Committee, London – Westminster. REC reference 14/LO/2313.

### Recruitment and selection of LHWs

All COPD patients who had completed PR classes in two PR services in the London Boroughs of Lambeth and Southwark within the previous 18 months were invited to express interest in training as volunteer LHWs. Suitable applicants were interviewed by two members of the research team (PW and GG) at which scenarios about confidentiality and maintenance of appropriate boundaries were used. An outline of the LHW training, a formal role description, and a volunteer agreement were provided before interview (Sections A “Role description for lay health worker” and B “Volunteer agreement” in Supplementary materials). Requirements of the role are given in Box 1.

### LHW training and mentoring

Recruited volunteers participated in a 3-day training course commissioned from the Royal Society of Public Health.^16^ Training objectives set in the commissioning process included the following: generic communication skills, confidentiality, role boundaries, and overcoming barriers to PR participation using specific behavior-change techniques (BCTs). The BCTs were selected to address barriers and facilitators to participation in PR.^9^ The BCTs were identified from the BCT taxonomy V1.^17^ The choice of BCT to tackle the barriers and facilitators was based on expert consensus regarding those most likely to influence these factors.^18^ The BCTs included goal-setting, problem-solving, social support, and information provision. For example, if patient-participants thought travel to PR would be a problem, the LHW could help with journey planning, suggesting family or friends to help with transport, or offer themselves to meet the patient on the way to PR.

Box 1 **Role requirements of the LHWs (full role description available in Supplementary materials)**

**Table:** 

**Volunteer LHWs were expected to**
**1. Attend LHW training**
Three sessions (10.30 am – 3.30 pm) 1 day a week for 3 weeks (lunch and refreshments provided).
**2. Undergo DBS**
DBS check is a criminal record check.
**3. Support patients newly referred for PR**
Each LHW to support between four and eight patients over a period of 6 months.
**4. Take part in a regular mentoring group**
To attend at least four mentoring meetings in the course of the project.
**5. Research activities**
To record as instructed telephone and face-to-face interactions with patients and to participate in a research interview as part of the study evaluation.

**Abbreviations:** DBS, disclosure and barring check; LHWs, lay health workers; PR, pulmonary rehabilitation.

The training was observed by a member of the research team (GG) noting subjects covered, methods used, achievement of goals, and testing of learning outcomes. Smartphones were provided to enable LHWs to keep their personal phone details private. A smartphone training session was included in day 2 of the training. The phones were used by the LHWs to record all their interactions with referred patients, both phone calls and any face-to-face meetings. All available recordings were transcribed verbatim to facilitate assessment of intervention fidelity.

Feedback was obtained at end of training by questionnaire and at the end of the study at interview. After the training course, LHWs met regularly with a specialist group mentor, an organizational psychologist, to discuss support of patient-participants, learnt techniques, and to receive peer support. Meetings were held monthly for the first 3 months then once every 2 months. At the end of the intervention, LHWs were invited to take part in a qualitative interview to assess their experience. A topic guide (Section C “Topic guide for lay health workers: end of study interview” in Supplementary materials) was used to guide the interviews. LHWs were offered payment for the research elements of the LHW role (recordings of interactions and a feedback interview): £50 per patient supported and £60 for the final interview.

### Recruitment of COPD patient-participants

Invitations to newly referred COPD patients to participate in the research were enclosed with PR appointment letters in three PR services in the London Boroughs of Lambeth, Lewisham, and Southwark. Nonrespondents to the mail and to a reminder were also followed up by telephone by the PR clinical teams. Interested patients met the research associate (GG) who obtained informed consent. Patient-participants’ contact details were given to an LHW within 3 days of the consent meeting. Patient-participants’ preferences for a male or female LHW were met if practicable.

The PR programs in the three London Boroughs consisted of an initial assessment of suitability followed by 2-hour classes twice a week for 7 or 8 weeks depending on the PR service. The components of the classes were individualized, progressive aerobic, strengthening and flexibility exercises, and education with an emphasis on improving self-confidence in disease management. An assessment at the end of the PR program included planning and signposting to local services to facilitate maintenance of increased levels of activity.

A self-administered questionnaire of patient-participants’ views was administered by PR teams at final PR assessment (Section D “Questionnaire for patients who have been supported by a lay health worker” in Supplementary materials). A copy was posted to patient-participants who did not complete PR or did not attend the final PR assessment. Two reminders were sent to nonrespondents. All respondents were invited to indicate their willingness to take part in a more detailed interview with a researcher. Face-to-face semi-structured qualitative interviews were undertaken. Interviews were guided by a topic list and took place at a venue convenient for participants. Most were completed at their home address. The interviews were recorded and transcribed verbatim. Respondents were recruited until thematic saturation was achieved. Patient-participants were offered remuneration (£50) for the research interview.

### Intervention delivery and fidelity

Intervention delivery and fidelity were assessed by the frequency and setting (telephone or face to face) of LHW–patient-participant pair discussions and through analysis of transcribed recordings. Fidelity of intervention delivery was defined as the extent to which the intervention was delivered as intended.^19^ Appropriateness of delivery style and fidelity of delivery of the intervention were assessed by two raters using a checklist based on the BCTs taught in the training sessions.^20^

### Analysis

Analysis of recruitment, training participation (LHWs only), retention, and questionnaire assessment was by descriptive statistics (participant characteristics, frequencies, means, and ranges). Analysis of the appropriateness of delivery and use of BCTs was based on a coding framework developed by AJW, GG, and PW. An analysis checklist was piloted on the transcribed interactions of three LHW–participant pairs, each with a different LHW and coded independently by three coders (AJW, PW, and GG). Discrepancies in coding were resolved by discussion. The amended checklist was tested by the three coders on a further three pairs.

To assess intervention fidelity and delivery style more fully, two coders (AJW and VmM – see acknowledgements) coded and analyzed 24 interactions, at least two interactions for each LHW that had been actively providing patient support. Interactions selected were the first recorded interaction by each LHW and a recorded interaction half-way through recordings by that LHW. Scoring of fidelity of delivery was based on evidence of adherence to taught BCTs and competence in applying them.^21^–^24^ Inter-rater reliability was assessed by examining the proportion of all BCTs identified within a transcript that were identified by both coders (ie, percentage of positive agreements).^25^

Qualitative data, from audio-recordings of interviews with LHWs and with patient-participants, were organized using NVivo 12 software (QSR International, Cheshire, UK) and analyzed thematically.^26^,^27^ These data will be reported separately in another paper.

## Results

### Recruitment of LHWs and patient participants

**Figure 1:**
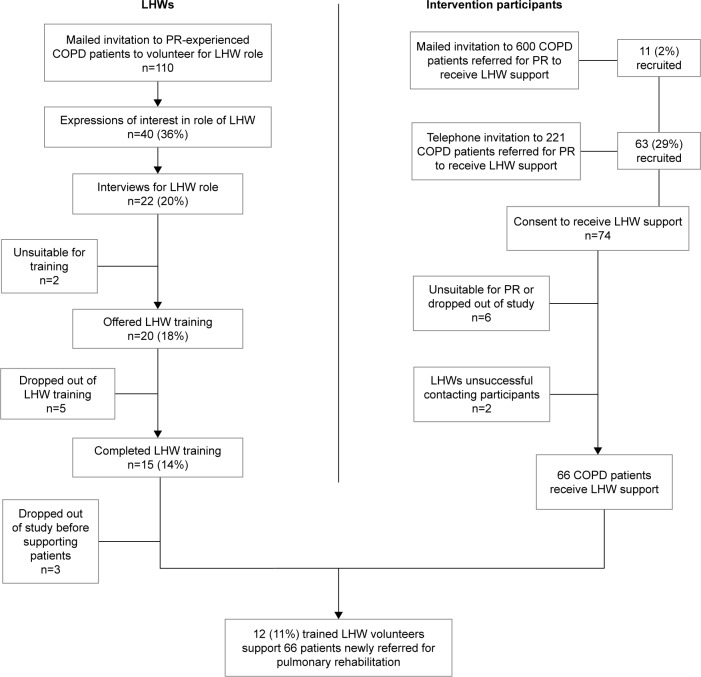
Recruitment flowchart. **Abbreviations:** LHWs, lay health workers; PR, pulmonary rehabilitation.

One hundred ten COPD patients eligible for the LHW role were contacted. Forty (36%) expressed interest, 20 (18%) were selected, and 15 (14%) completed LHW training. Twelve LHWs supported patients. The process of recruiting and selection of LHWs is shown in Figure 1. The characteristics of the LHWs are shown in Table 1.

Seventy-four COPD patients newly referred to PR were recruited to receive LHW support. Of these, eleven were recruited via a mailed invitation (2% of the 600 who were mailed an invitation). Sixty-three were recruited by telephone (29% of the 221 invited using this method). The process of recruitment of patient-participants is shown in Figure 1.

### LHW training and mentoring

Twelve trained LHWs took part in the intervention. The training was observed directly by GG. The first training session included orientation in a learning environment. Over the 3 days of the course time was spent on communication skills, confidentiality, boundary setting, personal safety, use of a smartphone, and the learning and practice of BCTs. Role-play was used to build confidence in applying BCTs and to assess readiness to intervene with patient-participants. The pace of learning differed between LHWs. Assessment of learning outcomes was informal. It was apparent that more time was needed during training to assess the effectiveness of the learning. This was particularly relevant to applied learning and skill reflection in the use of BCTs which was not tested. LHWs had varying ability in using the phones. Three additional sessions on the use of a smartphone were provided (GG). The LHWs would have welcomed more personalized training in addition to the three group training days.

Eight mentoring meetings for LHWs were held throughout the intervention and the average attendance at these meetings was 8 LHWs (range 6–12). Mentoring meetings addressed barriers to promoting patient attendance at PR including difficulties in contacting patients and overcoming patients’ travel barriers to attendance.

### Delivery of the LHW intervention

Three trained volunteers withdrew from the program. One was unable to master use of the smartphone, one suffered a bereavement, and one had a family crisis. There was a gap of up to 3 months between the training of LHWs and the first recruitment of patients for them to support. This was due to low response of patient-participants to mailed invitations (Figure 1). The introduction of phoned invitations was followed by an extension of the intervention period to 10 months. Eight patient-participants made requests for specific gender LHWs and all were allocated to the LHW gender requested. Six (8%) patient-participants dropped out of the study after recruitment but before contact with an LHW and the LHWs were unable to contact two patients. Sixty-six patient-participants took part in the intervention. One patient-participant left the area during the research and could not be contacted for follow-up.

Sixty-six COPD patients were supported by LHWs, 5.5 patients per LHW (range 3–8). Recordings were made in 60 LHW–patient-participant pairs. In six pairs, interactions were not available due to recording problems. One LHW lost his phone for 3 weeks. All LHWs provided phone support to patient-participants. Nine LHWs also had face-to-face meetings with patient-participants.

Three hundred and twenty-nine pair interactions were recorded and transcribed. LHW–patient pairs had 5.4 interactions on average. Some had frequent and prolonged contact over a 2 to 3-month period. In two pairs, there were 20 or more interactions. Two patient-participants were transferred to a different LHW during the intervention due to family problems experienced by two LHWs. Ten LHWs were retained to the end of the study. Four LHWs had periods of illness during which they could not deliver the intervention, two due to COPD exacerbations.

### Intervention fidelity

The quality of communication between LHWs and patient-participants was high. There was abundant evidence of the use of the BCTs taught in training, with all of the LHWs using at least some BCTs. The most widely used BCTs were information provision about PR and its benefits and provision of social support. The delivery style of BCTs was appropriate. However, attempts by LHWs to identify each patient-participant’s barriers or facilitators to PR were limited. This meant that, in those patient-participants, LHWs were limited in their tailoring of the BCTs delivered. Detailed analysis of intervention fidelity will be reported separately.

### Acceptability of LHW support to COPD patients

Forty (62%) COPD patient-participants who received LHW support completed a questionnaire. Thirty-four (85%) were very satisfied or satisfied with their first LHW contact. Twenty-nine (73%) were very satisfied or satisfied with most areas of contact. Five respondents who had not had regular contact with the LHW left most options blank. In four of these LHW–patient pairs, the LHW was not in touch with the participant regularly due to LHW ill health. No patient-participants commented at interview on the additional burden of participating in this research.

## Discussion

Recruitment, selection, and training of PR-experienced COPD patients as LHWs to improve the uptake and completion of PR is feasible. The rate of recruitment of LHWs was high and LHWs understood and accepted the importance of breach of confidentiality and of interpersonal boundaries.^12^ Appropriate delivery and limited fidelity of the intervention were demonstrated. Almost 30% of the newly referred COPD patients accepted telephone invitations and participated in the research. This is a high response rate in patients with substantial illness and average failure of uptake of PR of 20%–30%. Mailed invitations were ineffective. LHW support was perceived positively by patient-participants. A cluster randomized controlled clinical trial of the LHW intervention is deliverable within acceptable cost limits.

This research presents a new form of lay or community health working. The LHW training was bespoke, based on a health psychology approach to mapping the determinants of PR uptake and completion.^9^,^18^ Pitching the LHW training at the right level was challenging because of the education team’s lack of previous experience of teaching BCTs and the lack of evidence on effective comparable training in the literature. The approach to the assessment of intervention fidelity in this study, using smartphones to record all interactions in LHW–patient pairs, is novel and worked well. There was no evidence that LHWs were discouraged by the burden of training or that they or participant-patients were discouraged by the demands of the research.

### Strengths and weaknesses

LHWs undertook the joint volunteer and research participant roles effectively. Our patient advisory group, comprising PR-experienced COPD patients, felt that the voluntary status of the LHW was a key element.^28^,^29^ They felt that the community basis of the concept and the cooperative nature of PR itself could be undermined if the LHWs were not volunteers. Participants’ expenses were reimbursed. Payments for research participation were made at the stipulation of the funder. LHWs accepted the recording of all their interactions with patient-participants. The optimal level of ongoing supervision required when LHWs are established in routine practice remains to be determined.

Ongoing illnesses, both exacerbations of COPD and other comorbidities, were a problem for some patient-participants, as they were for LHWs. These illnesses led to difficulties of continuity for patient-participants and for LHWs, an issue to be addressed in planning a trial, and that has been factored into the trial sample size calculations.

The gap of 3 months between the training of LHWs and the recruitment of patient-participants may have affected LHWs retention of elements of their training. Inconsistent support by some LHWs of patient-participants and inadequate reinforcement in mentoring may have reduced the intervention’s effectiveness. These limitations will require adjustment in the design of a clinical trial. The LHWs did not always fully elicit individual patients’ barriers to and facilitators of PR uptake and completion. This suggests the need for greater emphasis and more time in training on tailoring the BCTs delivered to each individual’s salient barriers. The content of mentoring meetings should be reviewed to ensure reinforcement of BCT delivery. The model of recruiting patients, with experience of the target disease who have also undergone the specific treatment they are promoting, to be trained as LHWs is unusual, especially in health systems in high-income settings. A similar approach can be seen in low-income countries where individuals are recruited as LHWs to encourage patients, who share the same condition, to attend health care.^13^,^14^

## Conclusion

COPD patients experienced in PR can be trained as LHWs and can take on the role of promoting uptake and completion of PR among referred COPD patients. Newly referred COPD patients accept LHW support in a research setting, and trained LHWs can be retained in the program. Appropriate delivery and acceptable fidelity of the intervention were demonstrated. There is room for further development of the LHW training to focus more on tailoring the intervention to patients’ personal barriers to PR uptake and completion. More attention should be paid to ongoing supervision of LHWs as they move from training to actively delivering the intervention. Fidelity to the intervention requires reinforcement because LHWs are vulnerable to interruptions due to COPD exacerbations and to comorbidities. Interruptions due to illness should be considered in research planning, but they are minor adjustments in preparing for a full-scale clinical trial. Our evaluation suggests that a clinical trial of the LHW intervention in promoting PR for COPD is feasible. Work to plan such a trial is underway.

## Supplementary materials

### Role description for lay health worker

**Responsible Organization:** King’s College London, Division of Health and Social Care Research.

**Role Title:** Lay health worker in pulmonary rehabilitation.

**Responsible to:** Dr Patrick White, Clinical Senior Lecturer in General Practice and Primary Care.

**Purpose/Summary of the role:** To assist COPD patients newly referred to Pulmonary Rehabilitation in attending the course.

**Description of tasks**

1. To agree to support up to eight patients referred to pulmonary rehabilitation over a period of up to 12 months.
2. To attend three training sessions in the role of lay health worker.
3. To support the patient using the understanding and skills acquired at the training sessions.
4. To speak with, meet and, if desired, accompany the patient to pulmonary rehabilitation.
5. To make a digital recording, using equipment provided by the research team, of conversations with patients to help with evaluation of this project.
6. To attend meetings with other lay health workers arranged by a professional mentor. The purpose of the mentor meetings is to review the role of lay health worker. Lay health workers will be able to share experiences, to learn from each other, and to solve problems that arise.
7. To treat all personal information given by patients in complete confidence.
8. To inform the research team of any concern or worry about the lay health worker’s own welfare that arises in the course of the volunteering role.
9. To inform the research team of any concern or worry about the welfare of any patients that arises in the course of the volunteering role.
10. To provide an interview to the research team to evaluate the role of being a lay health worker.

**Time commitment:**

1. To agree to act as a volunteer for up to 1 year.
2. To attend three training sessions in East Dulwich.
3. To provide support for up to 8 patients, 2 months at a time (with some overlap) over a year.
4. To speak on the telephone with each patient no more than eight times. Each telephone conversation to take no more than 30 minutes.
5. To meet with each patient at least once and no more than four times. Each meeting to last no more than 3 hours.
6. To attend at least four mentoring meetings (1½ hours max each) in the course of the year.

**Skills and qualifications:**

1. Previous experience of at least one complete course of Pulmonary Rehabilitation for COPD.
2. Ability to speak, read, and write in English.
3. Ability to use a telephone.
4. Ability to travel independently in south London.

**IMPORTANT NOTE:** All volunteer lay health workers must undergo a Disclosure and Barring Service check, previously known as a Criminal Record Bureau check. We will provide more information on this at interview.

**Training and support:**

Training will be provided by the Royal Society of Public Health at Dulwich Community Hospital on 3 days (1 day a week for 3 weeks). Each session will be from 10.30 am to 3.30 pm. We will provide a mentor for lay health workers who will meet with them in a group and support them in their work with patients.

**Reimbursement of expenses:**

Travel and subsistence, will be reimbursed, each at a set rate to be agreed.

**Payment for research work:**

The volunteer will be paid for activities related to the research evaluation. These will include a payment of up to £60 per patient supported for the recording of conversations with patients and a one off payment of £50 for providing a research interview. Lay health workers who support eight patients will be paid £480 for their research contribution in recording interviews.

**Benefits to volunteer:**

Volunteering as a lay health worker is an opportunity to support other COPD patients to benefit from the pulmonary rehabilitation service. We hope the volunteer will find this role enjoyable and fulfilling. We think it will prove extremely valuable to patients. This is the first time support through lay health workers has been attempted in this setting.

### Volunteer agreement

Important Note: This agreement is not intended to be a legally binding contract of employment.

Volunteers are an important and valued part of clinical research carried out at King’s College London. We hope that you enjoy volunteering with us and feel a full part of our team. This agreement tells you what you can expect from us and what we hope from you. We aim to be flexible, so please let us know if you would like to make any suggestions and we will do our best to accommodate them.

**We, the Department of Primary Care and Public Health Sciences, King’s College London, will**

• Introduce you to how the research works and your role in it and provide any training you need.

- Provide regular contact with the lay health workers research team so you can tell us if you are happy with how your involvement is organized and get feedback from us.
- Respect your skills, dignity, and individual wishes and do our best to meet them.
- Keep you up to date with the progress of the research and inform you of possible changes directly affecting you.
- Provide a safe workplace.
- Apply our Diversity policy.
- Address concerns and issues you may raise to reach solutions via our problem-solving processes.

**I agree to**

- Have an ongoing obligation to inform the Department of Primary Care and Public Health Sciences, King’s College London, of any criminal charges, convictions, or cautions which occur during the course of my volunteering, whether or not they are related to the volunteer work. I understand failure to do so may result in disciplinary action or dismissal from the research team.
- Attend reliably at the time and place agreed and to give as much notice as possible whenever I cannot perform my role as expected.
- Follow King’s College London’s rules and procedures, including infection control, health and safety, diversity, and confidentiality.
- Raise any concerns about my experience as a volunteer at an early stage, giving the research team staff the opportunity to resolve any issues.
- Behave with courtesy to all colleagues, patients, and staff that I encounter in the course of my volunteering duties.

**Name:**

I accept the Agreement offered. In particular, I note my responsibilities in respect of Infection Control detailed in the Occupational Health form and confidentiality.

**Signed: Date:**

### Topic Guide for Lay Health Workers: end of study interview

The purpose of the session is for us to hear about the experience of the lay health workers in supporting patients newly referred for pulmonary rehabilitation.

It is important that you feel able to say negative things about the project and positive because both types of comments could benefit people with COPD in the future. There are no right or wrong answers, we recognize that involvement in this project is different for everyone – we just want to hear about how your experience went and your thoughts about it.

**Emphasize that everything said is confidential and will only be used for research purposes**

1. What attracted them about volunteering as a lay health worker?
  What were their expectations?
  Probe – things that they thought might be good about getting involved?
  Things that they were worried about being involved?
2. How did they find the process of volunteering – information provided, interview?
3. How did they find the 3-day training as preparation for the role – what strengths, what gaps?
  Probe – did they feel different by the end compared to at the start of the training?
4. How did they find the arrangements for the introduction of the lay health workers to patients?
5. How did they feel the patient support went? Anything that they can give as examples of things they felt went particularly well, or things that they felt could have gone better?
  Follow-up – How was the first call, how were they feeling?
  How was the time involvement?
  How did they find the recording of calls and conversations?
  Did they have to adjust their approach for different patients they supported?
6. Once they were supporting patients, what do they think were the key issues that influenced patients’ participation in pulmonary rehabilitation?
7. How did they find the monthly mentoring meetings?
8. What do they think about the reimbursement of expenses and the payment for research participation?
9. What additional arrangements would have made the process better? Or, if we were going to do this project again, what advice would you give us?

Overview of the idea of lay health worker’s involvement in the recruitment and retention of patients to pulmonary rehabilitation.

### This questionnaire is for patients who have been supported by a lay health worker as part of the Drill project

**IMPORTANT NOTE:** The information you provide in this questionnaire is for the research team. Any information you provide about your lay health worker and your comments will be treated as confidential.

**Name:**

**Your satisfaction with the lay health worker:**

**Table:** 

How satisfied were you with the contact you had with your lay health worker?				
	Please put a tick in one column for each			
	Very satisfied	Satisfied	Dissatisfied	Very dissatisfied
Initial phone call and introduction				
Face-to-face meeting(s)				
Subsequent telephone calls				
Ability to listen				
Ability to help with any problems going to PR				
Ability to answer questions and explain things				
Frequency of contact				
Quality of communication				
Overall support from lay health worker				
Overall satisfaction with lay health worker				

**Do you have any other comments about your lay health worker?**

**General comments about the Drill Project**

We will be interested to read your comments or suggestions about this new approach to supporting COPD patients to attend pulmonary rehabilitation.

**Please tell us what you think are the strengths of the Drill project.**

**Please tell us where you think the Drill project could be improved.**

**Figure:**
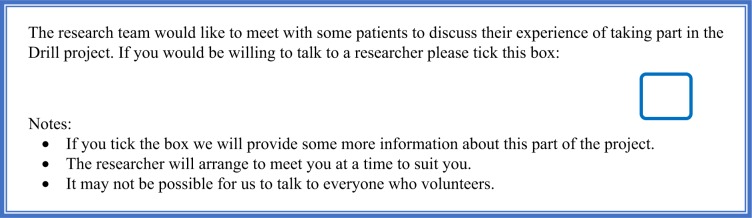


**Thank you for completing this questionnaire and for your participation in the Drill Project.**

## Figures and Tables

**Table 1:** Characteristics of volunteer LHWs who started training and of all patients invited to become LHWs

Characteristics	Volunteers accepted for training (n=20)	Patients invited (n=110)

Mean age (range)	67.5 (55–79) years	69 (43–89) years

Male (%)	11 (55%)	57 (52%)

Ethnicity		
White British	17 (85%)	88 (80%)
White other	0	11 (10%)
Black British	0	2 (1.8%)
Black other	1 (5%)	4 (4%)
Asian	0	1 (0.9%)
Other	2 (10%)	2 (1.8%) (Missing =2)

Mean FEV_1_ % predicted (range)	49% (28%–85%) Missing =3	56% (18%–102%) Missing =13

Mean time since completing PR (range)	5.5 (0–14) months	4.6 (0–14) months
PR venue		
Hospital setting	14 (70%)	65 (59%)
Community setting	6 (30%)	45 (41%)

**Abbreviations:** LHWs, lay health workers; PR, pulmonary rehabilitation.
